# Supplementary material for: Impact of COVID-19 pandemic related stressors on patients with anxiety disorders: A cross-sectional study
Source: PLoS One. 2022 Aug 18;17(8):e0272215. doi: 10.1371/journal.pone.0272215 (PMC9387803; doi:10.1371/journal.pone.0272215)
Supplement: S1 File — (DOCX) [file pone.0272215.s001.docx]

**Supplement**

**S1**

**Pre-pandemic anxiety symptoms.** Severity of anxiety symptoms before the pandemic was assessed with the Hamilton Anxiety Rating Scale (HAM-A; (1), a clinician-based questionnaire consisting of 14 items with sufficient internal reliability (Cronbachs alpha: .83-.87; 2). The queried symptoms include psychological and somatic complaints. Each symptom is rated on a Likert scale from 0 (not present) to 4 (severe) by the clinician. A sum score of less than 18 indicates mild, 18 to 24 mild to moderate, 25 to 30 moderate to severe, and more than 30 severe anxiety symptoms.

**Pre-pandemic depressive symptoms.** Pre-pandemic severity of depressive symptoms was measured with the revised version of Beck Depressive Inventory (BDI-II; (3), a well-validated self-assessment with 21 items based on the DSM-IV). Cronbachs alpha for the german version: depressive patiens in treatment (N = 288) .93, patients with another primary diagnosis (N = 123) .92, healthy subjects (N = 582) .90 (4). Each symptom is rated on a Likert scale from 0 (not present) to 4 (severe) by the clinician. A sum score of less than 18 indicates mild, 18 to 24 mild to moderate, 25 to 30 moderate to severe, and more than 30 severe anxiety symptoms.

**Modified Panic and Agoraphobia Scale.** The PAS (5) measures the severity of symptoms in patients with panic disorder with or without agoraphobia. It consists of 14 items. Apart from the total sum score, five subscales can be generated: panic attacks, agoraphobic avoidance, anticipatory anxiety, disability, and health concerns. The self-report version of the PAS shows good internal consistency (Cronbachs alpha: .85) and validity (correlation with similar questionnaires range from .58 - .91; 5,6). We omitted two items from the original questionnaire. One item is an additional item that is not included in the total score (“Did most attacks occur unexpected or expected [in dreaded situations]?”). Another one did not fit to the rating scale and could not capture a change in the severity of a specific anxiety symptom (“How important were the situations you avoided?”). Thus, the modified version of the PAS (in the following called PAS-m) consisted of 12 items (Cronbach´s alpha: .85).

**Modified Liebowitz Social Anxiety Scale.** The LSAS is a well-validated scale and a common instrument for assessing dimensional severity of symptoms of social anxiety disorder (7,8). The 24 items are divided into two scales: fear and avoidance of social situations. Furthermore, social situations are subdivided into social interactional situations and performance situations. Hence, apart from the LSAS total score, six subscale sum scores are provided: total fear, fear of social interaction, fear of performance, total avoidance, avoidance of social interaction and avoidance of performance. According to (8) the scales possess high internal consistency (Cronbach´s alpha between .81 and .96) and substantial convergent and discriminant validity. For the study described here, the items of the original questionnaire stayed the same. Only the rating scale was changed: In the original version, the items are answered on a 4-point Likert scale (from *none* to *severe* for the fear-scale and from *never* to *usually* for the avoidance scale). In our study, the rating scale aimed for potential changes in the symptomatology and thus, was modified as described above. We refer to the modified LSAS as LSAS-m (Cronbach´s alpha between .93 and .98).

**Modified DSM-5 Severity Measure for Specific Phobia – Adult.** The SMSP-A assesses symptom severity of specific phobia based on the DSM-5 criteria and has shown high internal consistency in previous studies (Cronbachs alpha: .83; 9). The ten items stayed the same in this study. We modified the original 5-point Likert scale from *never* to *all the time* as specified above and refer to this scale as SMSP-m (Cronbach´s alpha: .95).

**Convergent and divergent validity of the modified Questionnaires.** The modified anxiety scales show high correlations with each other whereas the correlation with PHQ are smaller or not significant (Tab S1).

**Table S1. Correlations.**

|  |  | **PHQ 15 – somatic symptoms** | **PAS - M** | **LSAS – M** | **SMSP - M** |
| --- | --- | --- | --- | --- | --- |
| **PHQ 15 – somatic symptoms** | r | 1 | 0.271 | .478** | .277 |
| **Step 1** | Sig. |  |  |  | .084 |
| Job worries (12) | N | 46 | 44 | 41 | 40 |
| **PAS - M** | r |  | 1 | .341* | .361* |
| **Step 1** | Sig. |  |  | .031 | .022 |
| Pre-pandemic depression | N |  | 45 | 40 | 40 |
| **LSAS – M** | r |  |  | 1 | .779** |
| Pre-pandemic depression | Sig. |  |  |  | .000 |
| Relationship worries (5) | n |  |  | 42 | 37 |
| **SMSP - M** | r |  |  |  | 1 |
| Pre-pandemic depression | Sig. |  |  |  |  |
| Relationship worries (5) | n |  |  |  | 41 |

**. Correlation is significant at the 0.01 level (2-tailed).

**S2**

In the original analysis, the Item divorced/separated was included in the final model. As this only applies to two subjects, this can be interpreted as an artifact. Removing all variables with at least one characteristic represented less than 10 % of all subjects before the analysis changes the results as compared to the original analysis as follows.

**Table S2. Summary of Hierarchical Regression Analysis for Variables predicting changes in anxiety symptoms and severity of depression.**

|  | β | *t* | sr² | *R* | *R²* | *∆R²* |
| --- | --- | --- | --- | --- | --- | --- |
| **Changes in anxiety symptoms** |  |  |  |  |  |  |
| **Step 1** |  |  |  | .48 | .23 | .23 |
| Job worries (12) | .48 | 3.65** | .23 |  |  |  |
| **Step2** |  |  |  | .59 | .35 | .13 |
| Job worries (12) | .38 | 3.05** | .14 |  |  |  |
| Pre-pandemic anxiety | .37 | 2.91** | .12 |  |  |  |
| **Severity of depression** |  |  |  |  |  |  |
| **Step 1** |  |  |  | .75 | .56 | .56 |
| Pre-pandemic depression | .75 | 7.57*** | .56 |  |  |  |
| **Step2** |  |  |  | .83 | .68 | .12 |
| Pre-pandemic depression | .67 | 7.67*** | .36 |  |  |  |
| Relationship worries (5) | .36 | 4.14*** | .12 |  |  |  |
| **Step 3** |  |  |  | .85 | .72 | .04 |
| Pre-pandemic depression | .62 | 7.25*** | .34 |  |  |  |
| Relationship worries (5) | .37 | 4.45*** | .13 |  |  |  |
| Work in health | .20 | 2.38* | .04 |  |  |  |
| **Step 4** |  |  |  | .87 | .76 | .04 |
| Pre-pandemic depression | .62 | 7.69*** | .34 |  |  |  |
| Relationship worries (5) | .40 | 5.09*** | .15 |  |  |  |
| Work in health | .26 | 2.85** | .05 |  |  |  |
| Meaningful work (16) | -.20 | -2.56* | .04 |  |  |  |

*N* = 47; **p* <.05. ***p* < .01. ****p* < .001.

1. Hamilton M. The assessment of anxiety states by rating. Br J Med Psychol. 1959;32:50–5.

2. Maier W, Buller R, Philipp M, Heuser I. The Hamilton Anxiety Scale: reliability, validity and sensitivity to change in anxiety and depressive disorders. J Affect Disord. 1988;14(1):61–8.

3. Beck AT, Steer RA, Brown G. Beck Depression Inventory–II. Psychol Assess. 1996;

4. Kühner C, Bürger C, Keller F, Hautzinger M. Reliabilität und validität des revidierten Beck- Depressionsinventars (BDI-II). Befunde aus deutschsprachigen stichproben. Nervenarzt. 2007;78(6):651–6.

5. Bandelow B. Panic and Agoraphobia Scale (PAS) manual. Seattle: Hogrefe & Huber Publishers; 1999.

6. Bandelow B. Assessing the efficacy of treatments for panic and agoraphobia. II: The Panic and Agoraphobia scale. Int Clin Psychopharmacol. 1995;10:73–81.

7. Liebowitz MR. Social Phobia. Mod Probl Pharmacopsychiatry. 1987;22:141–73.

8. Heimberg RG, Horner KJ, Juster HR, Safren SA, Brown EJ, Schneier FR, et al. Psychometric properties of the Liebowitz Social Anxiety Scale. Psychol Med. 1999;29(1):199–212.

9. Lebeau RT, Glenn DE, Hanover LN, Beesdo-Baum K, Wittchen HU, Craske MG. A dimensional approach to measuring anxiety for DSM-5: Dimensional measurement of anxiety for DSM-5. Int J Methods Psychiatr Res. 2012 Dec;21(4):258–72.
