# Supplementary material for: Impact of COVID-19 pandemic related stressors on patients with anxiety disorders: A cross-sectional study
Source: PLoS One. 2022 Aug 18;17(8):e0272215. doi: 10.1371/journal.pone.0272215 (PMC9387803; doi:10.1371/journal.pone.0272215)
Supplement: Attachment — Submitted filename: SfA_Langhammer_Response_220510.docx [file pone.0272215.s002.docx]

**Response to the Reviewers**

We thank the reviewers for this overall positive assessment and the detailed constructive comments on the individual parts of the manuscript. In addition, we have thoroughly revised the language of the manuscript.

**Reviewer #1**

**The authors investigated the impact of COVID-19 pandemic-related stressors on patients with anxiety disorders. Main limitation is a small sample size (49 patients). To find protective or risk factors for symptom severity for anxiety disorder, substantial changes in research methods are needed.**

Reply:

We agree with the reviewer that the sample size is only moderate. We added the following sentence to the limitations to ensure that the reader considers this limitation. However, in contrast to other COVID-related studies, we here provide clinical data from actual patients who underwent a gold standard structured clinical assessment with well-validated diagnoses compared to the majority of investigations using convenience samples with self-reported symptoms and without a comprehensive clinical assessment. We are therefore confident that our data has sufficient merit to legitimate publication despite the moderate sample size.

Line 369 ff.:

*“…Third, the small sample size in combination with a relatively large amount of predictors in the regression analyses reduces the interpretability of the results.”*

**The abstract should include more specific results, including actual values and percentages.**

Reply:

We added the requested values in the abstract. We hope this makes it more informative.

**Page 7
We contacted 19 patients from the waiting list and 89 patients in treatment via post in July 2020. Forty-nine patients (response rate = 45.37%) participated. We excluded two patients from the study because they did not have a diagnosed anxiety disorder.
- Patient selection is unclear. It should be clear how many patients were initially selected. Why were patients other than those with anxiety disorders included first?**

Reply:

Indeed, this wording may be misleading. We changed the text as follows:

Line 127 ff.

*“…We contacted all patients that received a diagnosis in our specialized unit for anxiety disorders in our outpatient clinic. 19 patients from the waiting list and 89 patients in treatment signed an informed consent during the diagnostic process and therefore were contacted via post in July 2020. Forty-nine patients (response rate = 45.37%) participated. We excluded two patients from the study because they did not have a diagnosed anxiety disorder and were mistakenly contacted during the recruitment (there were exceptionally in treatment for affective disorders).“*

**Reviewer #2**

**The study looks at the impact of the pandemic on patients with anxiety disorders. The study thus contributes to a better understanding of the impact on this already vulnerable group and to identifying appropriate therapeutic implications.
However, the manuscript has some weaknesses, which I would like to point out below with questions for the authors.

Introduction in general: It would be great for the importance of the manuscript if the authors emphasised again at the end of the introduction why the question is important/novel.**

Reply:

We followed this advice and changed a paragraph as follows:

Line 98 ff.

*“…Their study and others determine pre-existing mental disorders as a specific risk factor for symptom load in times of a lockdown. However, those studies used convenience samples without valid diagnostic procedures. For treatment purposes and prevention programs, findings need confirmation in a well-characterized clinical sample. In addition, a transdiagnostic comparison would be helpful to differentiate between different needs for different kinds of anxiety disorders.”*

**Line 116: why were 19 people from the waiting list contacted. Then 7 people were included in the calculations. Wouldn't it be important to exclude these persons, since they had no therapeutic support during this time and thus differ substantially from the other persons in terms of support during this time? How do the authors justify these 7 persons or why were these 7 persons included?**

Reply:

The reviewer raises an important point. As the access to treatment was very limited during the acute phase of the pandemic, we expected both groups to be affected by the effects of the pandemic. We included the variable treatment (yes/no) in the regression model to test for the impact of treatment. The variable was not included in the final regression model and therefore did not show incremental validity. Potential limitations are discussed.

**Line 141: I am still not entirely clear whether the individual domains of the stressors should be considered coherently or whether the items simply stand alone and thus a statement on reliability is not necessary.**

Reply:

We only used the domains as a heuristic during the item construction and for the interpretation.

**If one looks at the individual items, then for some questions it could also lead to an item having to be answered with "does not apply" (e.g. “worried about job situation” cannot be answered if one is already unemployed. “schooling of my child” cannot be answered if one does not have a child). Was there such an answer category? if not, how do the authors deal with the fact that some items, which are also later in the regression, could possibly not be answered correctly?**

Reply:

Indeed, this consideration also guided the design of the survey and the adaption of the questionnaires. Therefore, participants had the possibility to choose “does not apply” per item. We defined this choice in our analyses as a missing value.

**Line 149 ff: can the authors say something about the validity and reliability of the adapted questionnaires? The answer category was changed for this study and it would be good for the reader if there were more psychometric information.
This is especially important for the PAS-m, where 2 items were also removed.**

Reply:

We have now added the requested information in the supplement. Here you will find Cronbach's alpha for the modified questionnaires and indices of convergent validity (correlations of anxiety measures with each other) as well as indices of divergent validity (correlations of anxiety measures with PHQ 15 for somatic symptoms). We did not evaluate any constructs that deviate more strongly from fear than the PHQ-15.

The two items left out from the PAS did not indicate the severity of panic symptoms as one is asking for situation associated with panic attacks and its importance (a list with multiple answer options) and the second one is asking if panic attacks were either mostly expected or unexpected.

Modified Panic and Agoraphobia Scale.

- Previous studies: Cronbachs alpha: .85
- Current study: Cronbach´s alpha: .85

Modified Liebowitz Social Anxiety Scale.

- Previous studies: Subscales - Cronbach´s alpha between .81 and .96
- Current study: Subscales - Cronbach´s alpha between .93 and .98

Modified DSM-5 Severity Measure for Specific Phobia – Adult.

- Previous studies Cronbachs alpha: .83
- Current study Cronbach´s alpha: .95

For more information see supplement.

**Line 222 ff: it is not clear to me how the 23 stressors were included in the regression. The figures show only 4 at a time and it is not clear from the text how they were chosen and also why this order was chosen e.g. in the first regression one would expect “pre-pandemic anxiety” to be included first.
If all 23 predictors were included, this is also not permissible due to the sample size.
It would be imperative for the understanding and interpretation of the results if the authors would present this regression in a little more detail or explain how they proceeded here (which predictors, why this order).
It would also be very welcome if it were clear to the reader exactly which predictors in the regression correspond to which items from Figures 3a and 3b. This is not possible at the moment.**

Reply:

Thank you for the very helpful comment.

We have changed the text as follows in order to be more precise in communicating the regression approach. We used a forward stepwise selection approach for our regression. Since this approach starts with zero regressors and includes only regressors with incremental validity based on statistical values (entry .05 and remove .10), this means that the highest number of regressors in the analysis is four in the final model.

In addition, we changed the table and figures so you can identify which items correspond to the variables in the regression.

*Methods:*

Line 188 ff.

*“…For the second hypothesis and testing for further protective and risk factors, we conducted two forward stepwise regression analyses. The first one tested the hypothesis that COVID-19 related stress (23 items), demographic (marital status, education, sex and age) and clinical characteristics (pre-pandemic anxiety and depression) predict changes in severity of the anxiety symptoms. The second one tested the same hypothesis for depressive symptoms.”*

**Line 258/259: I am not sure how profound this statement is, as only 2 people were divorced/separated at all. This has already been included in the limitations, but this seems insufficient to me under the fact that it is so small, as it is strongly shown as a protective factor here.**

Reply:

Thank you for this valuable suggestion. We have now added an exploratory analysis to the supplement. In this exploratory analysis, we excluded items if only very few participants answered differently. We set the cut off at 10%. So all dichotomous items with fewer than 4 participants on one side (≈ 10%) were disregarded.

*Results:*

Line 235 ff.

*“…, We exploratory repeated the analysis after excluding all dichotomous items with less than 10 % of the participants answering the less likely characteristic (see supplement).”*

Line 264 ff.

*“…In the exploratory analysis, the item divorced/separated was excluded and as an effect of this overextension with caring/schooling of children was not included after the first two steps of the regression.”*

*Discussion*

Line 370 ff.

*“…In our explorative analysis, two predictors, namely being divorced/separated and overextension with caring/schooling of children did not play a role in the best fitting model anymore. These variables should be interpreted with caution.”*

**Line 342 ff: The small sample size seems to be a limitation as well as the fact that it is a sample that was already in treatment. It is important to mention this, especially because the authors point out the practical implications that treatment options should be provided. However, this sample was in treatment and the authors should discuss again in detail what effect the treatment had on the increase. Did the therapists already react to this in treatment? How long were the individuals in treatment for, or how often were there therapy sessions during this time, or was therapy interrupted and this could have been an explanation for the increase?**

Reply:

Thank you for this thoughtful comment, which also relates to the second point about being in treatment vs. waitlist above. During the first wave of the pandemic, treatment options were very restricted, as digital treatments were not yet established in a regular manner. As such, treatment mode and dose is not representative of typical treatment at our outpatient center in pre-pandemic times. We now list the number of treatment sessions during the time window investigated and used this in the regression analysis. We changed the paragraph and added more information. In general, we were interested in symptom changes in diagnosed patients. It was a pragmatic decision to include both those in treatment and those on the waiting list.

Due to different confounding effects between the period of the last measurement before the pandemic and during the pandemic (e.g., number of perceived sessions varied from the therapy just started and more than 20 sessions), we decided to measure changes directly and not indirectly (in a pre- post manner). This way we hope to have found a pragmatic compromise. Your questions brought us to the decision of a direct measurement instead of indirect measurement. We did not measure specific reactions of therapists during this time. Also, we did not take possible interruptions into account as we wouldn’t expect worsening of symptoms through therapy disruption alone.

We changed the paragraph as follows:

Line 353 ff.

*“…Second, although pre-pandemic information about the symptom severity was available, a direct comparison of pre-pandemic and pandemic symptom severity was not meaningful. The period between measurement of pre-pandemic symptom severity and pandemic onset varied substantially. As most of the participants were in treatment this period was confounded with possible symptom changes through treatment. Still, we believe it is a straightforward approach to include both, those in treatment and patients on the waiting list as the pandemic affected the treatment and being on the waiting list. Treatment in some cases switched to online sessions or was disrupted for a longer period because of the restrictions and missing resources for online sessions. Still, numbers of sessions that varied extensively did not contribute to changes in our statistical model. In some way, the worsening of symptoms seemed to be independent of treatment as usual. Specific interventions in times of crisis seem necessary.”*

**Minor comments:
In line 76: would it be possible to find a more recent source?**

Reply:
We added a newer citation following this sentence as follows:

Line 80 ff.

*“…In a more recent review, the authors concluded that anxiety has a negative effect on depression in terms of symptom worsening, suicidal thoughts, and long-term outcome (36).”*

36.	Goldberg D, Fawcett J. THE IMPORTANCE OF ANXIETY IN BOTH MAJOR DEPRESSION AND BIPOLAR DISORDER: Importance of Anxiety in MDD and GAD. Depress Anxiety. 2012 Jun;29(6):471–8.

**Lines 79, 83: According to the journal's guidelines, is the year given in this form of citation? Otherwise, only a number is given here.**

Reply:

That is correct, thank you very much for catching it.

**Line 90: Here it would be helpful to know in whom higher rates were found.**

Reply:

We changed the sentences as follows:

Line 96 ff.

*“…On the one hand, they found higher rates in COVID-19 fear, depression and anxiety in patients. On the other hand, they observed a greater decrease of symptoms over time in patients versus non-patients.”*

**Line 108/109: it would be great for understanding if the German designation could be translated.**

Reply:

We included a footnote.

**Line 194: it would also be interesting to know the burden of the patients at the time of admission with regard to anxiety symptoms in order to get an idea of how burdened the persons were.**

Reply:

The average HAM-A score at the time of admission was 22.47 in which we now added in table 1. Values between 18-24 points indicate a mild to moderate severity of the anxiety disorder (see Hamilton, 1959).

**Table 1:
Under the subcategory "Employment status", was there also a question about working in the "health sector" or did this answer come from the stressors?**

Reply:

We added this information together with the stressor assessment as this is not routinely asked for at the time of admission in our outpatient clinic.

**Under the upper category "Domiciliary status", only the category "with partner" and "with children" was possible, or could one also tick "with partner and children"?
Under this category, 100% does not come up either.**

Reply:

Patients could choose multiple options or nothing in this category. Therefore, some left the field empty.

**In the upper category SES, the last line says (3.39%). Something seems to have slipped here.**

Reply:

For some variables, the table shows mean and standard deviation. We included more information in the table on that. The 3.39 is a standard deviation of the mean of SES and not percentage.

**Figure 2:
The first wave was already in March/April and therefore already over in July. It is therefore difficult to say that this was the symptom severity during the first wave (as described in the title of the figure). In July, the incidences had already been very low.**

Reply:

Thank you for your question. We changed the title into “...during and after the first wave...”.

„If you are asked about the time during the COVID-19 pandemic, please try to refer your answers to the **period since mid-March 2020** (nationwide closure of schools and numerous shops and facilities). If your experience and behavior has fluctuated over the period, try to assess how it was **on average**.” (Original instruction translated by the authors)

**Reviewer #3**

**The manuscript "Impact of COVID-19 pandemic related stressors on patients with anxiety disorders: A cross sectional study" presents an interesting investigation in which the effect of the first wave of the COVID-19 pandemic on stress, depression and anxiety in patients diagnosed with different pathologies (panic disorder, social phobia and specific phobia) is analyzed. The results show a positive association between stressors and symptom load.**

**The introduction is correct; please add the following reference (page 3 line 33) which discusses the change in emotions that occurred in the first wave comparing two groups and points out the increase in depressive states due to the pandemic: Meléndez, J. C., Satorres, E., Reyes-Olmedo, M., Delhom, I., Real, E., & Lora, Y. (2020). Emotion recognition changes in a confinement situation due to COVID-19. Journal of Environmental Psychology, 72, 101518. https://doi.org/10.1016/j.jenvp.2020.101518**

Reply:

Thank you for this valuable suggestion. We are happy to include this reference.

**Measurements. Line 127. the authors note that: “For the latter, we changed the instructions of well-established psychometric tests for anxiety symptoms in a way that we ask for changes instead of intensity of symptoms.” It might be interesting for the authors to include an example of the modification.**

Reply:

In the paragraph for “changes in anxiety symptoms”, we indicate the changes of the likert scales (including the exact words translated into English). We didn’t change the items itself but only the likert scales. Still, we added more information in the supplement and added a sentence as follows:

Line 145 ff.

„*...For more detailed information and psychometrics of the questionnaires used see supplement 1.”*

We also added:

Line 166 ff.

*“.... For most items, we only changed the dimension of the Likert scale. We had to change some items grammatically for a meaningful adaptation of the dimensions of the Likert scale.”*

**Please provide more information on the Beck Depressive Inventory (Similar to Hamilton: internal reliability, scoring scale, cut-off points...).**

**Please also provide information on how the Hamilton is scored: for example, "Each item is scored on a scale of 0 (not present) to 4 (severe)".**

**Please add these characteristics to all scales used in the manuscript.**

Reply:

We included all the missing information asked in the supplement.

**Although it is not necessary to include it in the manuscript, a question that arises in my mind is Why you did not use the same scale to assess depression during pre-pandemic and during pandemic? (BDI-II vs PHQ-9)**

Reply:

The reviewer raises an important point. We selected the PHQ-9 in order to make sure the current results can be compared to a second and larger study in indicated prevention during COVID-19 in the general population, which was at the same time conducted at our research group. Therefore, BDI-II scores are available from the pre-pandemic diagnostic assessment characterizing the sample, but PHQ-9 scores were (in line with the parallel investigation) used to asses changes. The methods paper of this sister-project is already published (Langhammer et al., 2021); the first results are currently under review (Hilbert et al., in press).

Langhammer, T., Hilbert, K., Praxl, B., Kirschbaum, C., Ertle, A., Asbrand, J., & Lueken, U. (2021). Mental health trajectories of individuals and families following the COVID-19 pandemic: Study protocol of a longitudinal investigation and prevention program. *Mental Health & Prevention*, *24*, 200221. <https://doi.org/10.1016/j.mhp.2021.200221>

Hilbert, K., Boeken, O., Seeman, S., Langhammer, T., Praxl, B., Horvath, L., C., Ertle, A., Asbrand, J., & Lueken, U. (under revision). Who is seeking help for psychological distress associated with the COVID-19 pandemic? Characterization of risk factors in 1269 participants accessing low-threshold psychological help.

**Regarding the modified questionnaires, I understand that what you have done is clinically correct, it is a good idea. However, it would be interesting if you could provide, if possible, information on the reliability of the modified questionnaires, this data would ensure that the modification would have been statistically correct.**

Reply:

Thank you for this important point which was also raised by reviewer 2. We have now added additional information in the supplement. Here you will find Cronbach's alpha, convergent and divergent validity for the modified questionnaires. Please also see response to comment X from reviewer Y.

**In data analysis (page 9 line 166) you report: first hypothesis ..., second hypothesis. Please, when you state your research objectives at the end of the introduction, could you please match this numbering of hypotheses.**

Reply:
We followed your advice and changed the text in the introduction.

**The results are correct. The explanatory figures provide the information in a satisfactory way and the regression analyses include all the necessary information.
The conclusions are correct and in accordance with the results.**

Thank you very much for your appreciation and the very constructive and helpful feedback.
