# Supplementary material for: Impact of COVID-19 pandemic related stressors on patients with anxiety disorders: A cross-sectional study
Source: PLoS One. 2022 Aug 18;17(8):e0272215. doi: 10.1371/journal.pone.0272215 (PMC9387803; doi:10.1371/journal.pone.0272215)
Supplement: Attachment — Submitted filename: Response to Reviewers.docx [file pone.0272215.s003.docx]

**Response to the Reviewers**

Again, we thank the reviewers for this overall positive assessment and the detailed constructive comments.

**Reviewer #2**

1) **The authors have stated that they have calculated a regression. My question at this point is: you included about 30 variables in the regression with a sample size of N = 47. This seems to me to be a very exploratory approach and my question is whether this number of variables is statistically justified?**

Reply:

We agree with the reviewer that the sample size is only moderate. However, in contrast to other COVID-related studies, we here provide clinical data from actual patients who underwent a gold standard structured clinical assessment with well-validated diagnoses compared to the majority of investigations using convenience samples with self-reported symptoms and without a comprehensive clinical assessment. We are therefore confident that our data has sufficient merit to legitimate publication despite the moderate sample size due to the high quality of clinical characterization.

Indeed, as the pandemic represented a very novel and unique situation, the regression analysis should be perceived as exploratory. Normally we would use theoretical knowledge based on previous research to reduce the number of variables included in the analysis. In this specific situation, however, our intention was to investigate the dynamics between pandemic-related stressors and pathological anxiety in a more explorative manner. As such, the relationship between predictors and sample size may bear a certain risk of overestimation

We therefore now added the following sentence to the limitations to ensure that the reader considers this limitation.

Line 369 ff.:

*“…Third, the small sample size in combination with a relatively large amount of predictors in the regression analyses may bear a tendency towards overestimation of explained variance.”*

**Additionally, it would be great if in the methods section under statistical analyses it is still described that only significant variables are presented and all others are omitted here. It would also be interesting to know in which step which variable was included.**

Reply:

As we used a stepwise regression model, the order of variables is not relevant in this hierarchical regression model. In contrast, the order is based on the process's algorithm, which selects variables based on statistical estimates.

2) **You wrote in your hypotheses/aims at the beginning (Introduction section) that you want to include health concerns, financial worries and pre-pandemic symptom severity as well as some socio-demographic variables to answer your question (regression analyses). But in the regression you find mainly variables of COVID-related stressors (number 12, 13, 5 and 16), which are not named in the hypothesis. Here I would like it to be clearer why the authors included these stressors in the analyses but did not mention them as variables to reach the aim at the end of the introduction.**

Reply:

Thank you for this thoughtful comment. In fact, the variables of interest in the hypotheses subsume different aspects represented by the different items. The relationship between the variables and the respective items is shown in Figure 3a. In addition and for the sake of clarity, we specified the text in the methods as follows:

Line 157 ff.:

*“Assessment of pandemic-related stressors. Patients completed questions on four domains: health concerns (5 items), social isolation (3 items), financial worries (4 items), and family- and work-related stress (11 items) based on a 5-point Likert scale (ranging from not at all to a lot/very much) or with yes or no. For details and assignment of items to categories, see Fig 3.“*

Figure 3a

Figure 3b
